# Identification of a Tick Midgut Protein Involved in *Babesia bovis* Infection of Female *Rhipicephalus microplus* Ticks

**DOI:** 10.3390/microorganisms13081713

**Published:** 2025-07-22

**Authors:** Sadie Izaguirre, Janaina Capelli-Peixoto, Rubikah Vimonish, Karen C. Poh, Sara Davis, Kierra Peltier, Kelly A. Brayton, Naomi Taus, Chungwon Chung, Massaro W. Ueti

**Affiliations:** 1 Program in Vector-Borne Disease, Department of Veterinary Microbiology and Pathology, College of Veterinary Medicine, Washington State University, Pullman, WA 99164, USA; sadie.izaguirre@wsu.edu (S.I.); j.capellipeixoto@wsu.edu (J.C.-P.); r.kirubananthan@wsu.edu (R.V.); kierra.peltier@wsu.edu (K.P.); kbrayton@wsu.edu (K.A.B.); 2 Animal Disease Research Unit, United States Department of Agriculture–Agricultural Research Service, Pullman, WA 99164, USA; karen.poh@usda.gov (K.C.P.); sdavis4@wsu.edu (S.D.); naomi.taus@usda.gov (N.T.); chungwon.chung@usda.gov (C.C.)

**Keywords:** *Rhipicephalus microplus*, midgut, *Babesia bovis*, bovine, protein, RNA interference

## Abstract

*Rhipicephalus microplus* is an important biological vector as it transmits several pathogens, including *Babesia bovis*, the causative agent of bovine babesiosis. The available strategies for controlling *B. bovis* are limited, resulting in substantial challenges for both animal health and livestock management. Infection of the tick midgut is the essential first step for the transmission cycle of *B. bovis*, yet this process remains largely unexamined. To better understand the first step of tick infection, this study employed a proteomic approach to identify a midgut protein that responds to *B. bovis* infection. We then used RNA interference for gene silencing to determine if the protein is essential for *R. microplus* infection. The protein we identified, Rm24, is twofold upregulated in the tick midgut during *B. bovis* infection. We silenced the gene encoding Rm24 and examined the effect of reduced expression on both tick fitness and *B. bovis* infection. Our results indicated that silencing the Rm24 gene impacted the survivability of adult female ticks, which exhibited a significant reduction in viability as compared to the control and non-injected groups. Importantly, we found that suppressing the gene encoding Rm24 led to a significant decrease in the number of engorged female ticks infected, with only 15% of female ticks testing positive for *B. bovis* kinetes as compared to over 50% in the control groups. We also detected a significant reduction in vertical transmission of *B. bovis* to larval progenies. These findings suggest that the Rm24 protein is critical for infection by *B. bovis* and could serve as a promising target for future transmission-blocking strategies.

## 1. Introduction

Pathogen–vector interactions remain a critical point in understanding the biology of pathogens and disease transmission. Many of these interactions are understudied in the arthropod vector but have notable effects on vector physiology and vector competence. *Babesia* parasites have co-evolved with their arthropod hosts to further develop and reproduce within the tick [1]. This relationship exposes potential untapped targets for novel therapeutics that may exploit these *Babesia*–tick interactions for transmission control.

Cattle fever ticks, specifically *Rhipicephalus microplus*, are one-host ticks, meaning that the larva, nymph, and adult stages all feed on the same animal [2]. Each stage of the tick requires a blood meal from an animal to complete its life cycle. When ticks feed on *Babesia*-infected animals, the ticks acquire the infection. After becoming fully engorged, the female ticks detach from the bovine and drop to the environment. The temperature change, along with other environmental stimuli within the midgut, triggers the *Babesia* parasites to develop into gametes [3]. Subsequently, the gametes fuse to form an infectious stage that invades the midgut epithelial cells [4].

Inside the midgut epithelial cells, *Babesia* transforms into kinetes, which are subsequently released into the tick’s hemolymph. This developmental stage of *Babesia* coincides with the tick’s reproductive process. The kinetes then infect the tick ovaries, which allows them to be transmitted vertically to the egg stage [5]. When these infected eggs hatch, the larvae quest for bovine hosts on which to feed, transmitting *Babesia* parasites in tick salivary secretions. Animals that are infested by these infected larvae typically experience severe acute disease approximately 9–12 days post-larval attachment. The adult female ticks are the only tick stage that acquire *Babesia* infection, and their offspring transmit *Babesia* during blood feeding on cattle [6,7].

Currently, there are limited control strategies to prevent the spread of bovine babesiosis with the main approach of control involving the use of chemical acaricides. However, populations of acaricide-resistant *R. microplus* have been identified, which can circumvent acaricide treatments [8]. Additionally, vaccination efforts have not been successful in controlling ticks or preventing disease outcomes. A few tick midgut proteins, including the Bm86 antigen, have been tested to control *R. microplus*. The Bm86 antigen as a preventative for tick control was found to be efficacious; however, its success was highly dependent on individual farm practices and other tick management strategies such as surveillance or acaracide treatment [9,10]. Some transmission-blocking strategies have shown promising results in reducing the spread of *Babesia*, but further studies are necessary to validate their efficacy in field conditions [11,12,13]. The shortage of antigens for developing vaccines against either the tick vector or *Babesia* prompted this study, which aims to identify tick proteins associated with *B. bovis* infection in the midgut, a crucial step for *Babesia* transmission via tick vectors. Understanding the protein interactions between the tick midgut and *Babesia* could enhance control strategies for bovine babesiosis.

## 2. Materials and Methods

### 2.1. Animals, Ticks, and Pathogen

In this study, four Holstein calves were utilized, including two spleen-intact calves (C1795, C1903), to generate uninfected ticks and two splenectomized calves (C1801, C1896) to produce infected ticks. The calves were age-matched between 3 and 4 months. They were cared for according to protocols approved by the University of Idaho Institutional Animal Care and Use Committee (Approved Protocol: IACUC-2024-09). Ticks used in the study were sourced from the laboratory colony of *Rhipicephalus microplus*, La Minita strain, which is maintained at the University of Idaho Holm Research Center in Moscow, ID. The *B. bovis* strain employed in these studies was the Texas strain which had been passaged twice in splenectomized bovines to produce stabilates referred to as the T2Bo strain for experimental infection.

### 2.2. Proteomic Analysis of R. microplus Female Ticks Exposed to B. bovis

We applied *R. microplus* larvae that hatched from 0.5 g of egg mass to a naïve spleen-intact calf (C1795) using a stockinette patch. The adult female ticks were collected and categorized as uninfected ticks. We applied another group of *R. microplus* larvae that hatched from 0.5 g of egg mass on another splenectomized calf (C1801) under a stockinette patch. Approximately 13 days after the application of the larvae, the calf was inoculated with 10^7^ *B. bovis* T2Bo organisms in a blood stabilate to synchronize peak parasitemia with adult tick acquisition feeding. Engorged female ticks that fed during *B. bovis* peak parasitemia were collected and categorized as infected ticks. The engorged female ticks collected from both infected and uninfected animals were held for four days post-engorgement before undergoing midgut dissection. Samples from individual ticks were flash-frozen in liquid nitrogen. Two groups of four ticks (*B. bovis* exposed tick group and unexposed tick group) were used to determine protein expression. For protein lysate preparation for mass spectrometry analysis, individual midguts were treated with a protease inhibitor cocktail using mini cOmplete Protease Inhibitor Tablets (Roche, Porterville, CA, USA), washed, and subsequently resuspended in RIPA lysis buffer (Thermo Fisher Scientific, Waltham, MA, USA) along with the protease inhibitor cocktail. The samples were sonicated six times for 15 s in an ultrasonic water bath (Thermo Fisher Scientific) and then centrifuged at 12,000× *g*, 4 °C for 15 min. The supernatant was removed and protein concentration was determined using the Pierce BCA Protein Assay Kit (Thermo Fisher Scientific). We standardized the concentration of proteins to a precise 1 mg/mL for each individual sample, ensuring optimal consistency and reliability in our results. Mass spectrometry experiments were performed at the Tissue Imaging, Metabolomics and Proteomics Laboratory at Washington State University in Pullman, WA using an Easy nanoLC 1000 UHPLC system coupled to a Fusion Orbitrap Tribrid mass spectrometer. To minimize bias in sample preparation and to account for technical variability in each sample, the raw mass spectrometry data were normalized using the Proteome Discoverer version 2.2 (Thermo Fisher Scientific) workflow, which uses a total peptide amount approach for normalization. Protein abundance means from the normalized and scaled abundances were compared using a Student’s *t*-test in Microsoft Excel, filtering to include only those entries with SEQUEST scores greater than 100 and percent coverage scores above 50. Initial protein characterization of midgut proteins was completed using InterPro 103.0, https://www.ebi.ac.uk/interpro/ (accessed on 3 April 2025) and AlphaFold https://alphafold.com/ (accessed on 3 April 2025) [14,15,16].

### 2.3. Synthesis of Double Stranded RNA

To assess gene function in tick infection, we performed an RNA interference (RNAi) experiment. The nucleotide sequence of Rm24 (NCBI Reference Sequence XM_037427095.1, derived from an *R. microplus* genome, NC_051172.1 [17] was analyzed using the E-RNAi web-based software developed by the German Cancer Research Center [18] to identify the target sequence that generated the highest number of small interfering RNAs (siRNAs). A template sequence was generated using PCR with a T7 promoter sequence attached to the 5′ end of each primer. The resulting 488 bp PCR product was purified using the GeneJET PCR Purification Kit (Thermo Fisher Scientific). Next, the MEGAscript™ RNAi Kit (Ambion, Austin, TX, USA) was used to transcribe the purified PCR product to produce double-stranded RNA (dsRNA). Additionally, a non-tick-specific control dsRNA was synthesized using a fragment (514 bp) from the *Drosophila melanogaster nautilus* gene (GenBank accession # M68897) as the target. The dsRNA was analyzed on a 1% nondenaturing agarose gel to confirm its integrity and to ensure the absence of single-stranded RNA (ssRNA) and double-stranded DNA (dsDNA). The purified dsRNA was quantified using a spectrophotometer (ND-1000, Thermo Fisher Scientific) and stored at −20 °C until needed.

### 2.4. Injection of Ticks with dsRNA

Larvae from approximately 0.5 g of tick egg mass were placed onto an uninfected calf (C1903) in a single feeding patch for rearing unfed adult ticks. Freshly molted adult ticks were collected on day 13 after the application of the larvae and then sexed, with the unfed female ticks being used for injection of the dsRNA. Unfed female ticks inoculated with Rm24-dsRNA were applied to a splenectomized calf (C1896) that had been inoculated with 10^7^ *B. bovis* T2Bo blood stabilate to synchronize peak parasitemia with female tick acquisition feeding. Three groups of 130 freshly molted, unfed female ticks were used in this study: (1) dsRm24 group (injected with dsRNA targeting the Rm24 transcript), (2) dsControl group (injected with non-tick-specific dsRNA), and (3) non-injected control group. Each tick was placed on adhesive tape to facilitate injection through the coxal membrane at the base of the fourth leg on the right ventral side. Ticks were injected with 1 µL of solution containing approximately 1 × 10^12^ molecules of dsRNA, using a 10 µL syringe and a 33-gauge needle (World Precision Instruments, Berlin, Germany). The injected female ticks were paired with male ticks in a 1:1 ratio and applied to an infected animal under separate stockinette patches. The adult ticks were applied approximately 7 days after the calf was inoculated with the *B. bovis* T2Bo strain. The infected calf was monitored daily for clinical signs of babesiosis, and samples were collected using peripheral blood to check for the presence of *B. bovis* parasites. Parasitemia was determined by qPCR targeting the ksp gene (BBOV_I002220), a single copy gene [19,20], which was quantified using a CFX Opus 96 Real-Time PCR System (Bio-Rad, Hercules, CA, USA). A standard curve for the analysis was generated by amplifying 10^0^ to 10^6^ plasmid copies of the *B. bovis* ksp gene. The qPCR reactions were performed using SsoFast™ EvaGreen^®^ Supermix (BioRad, Hercules, CA, USA), with three technical replicates at a final volume of 20 μL. The qPCR conditions were as follows: an initial cycle at 95 °C for 10 min, followed by 40 cycles of 95 °C for 15 s and 55 °C for 30 s. The specificity of the qPCR amplification was confirmed through melt curve analysis.

### 2.5. Gene Expression and Silencing

qRT-PCR was employed to assess gene expression and silencing of the Rm24 gene. *Rhipicephalus microplus* midgut tissue samples from 30 individual fed females were used per group to extract total RNA with the RNeasy™ Midi Kit (Qiagen, Germantown, MD, USA). Following extraction, the RNA was treated with DNase I (Thermo Fisher Scientific), and then 200 ng of RNA was used to synthesize cDNA with the SuperScript™ III First-Strand Synthesis SuperMix (Thermo Fisher Scientific). Primers targeting the Rm24 gene (Table S1) that avoided the region used for the construction of the dsRNA were utilized for the qRT-PCR. Primers (Table S1) targeting *R. microplus* glyceraldehyde-3-phosphate dehydrogenase (GAPDH), 40S ribosomal protein S3a, and elongation factor 1α (Elf1a) were employed as reference genes [21]. The qRT-PCR for all reference genes and the gene of interest was conducted on the CFX Opus 96 Real-Time PCR System using SsoFast EvaGreen™ Supermix (Bio-Rad) for detection. The cycling conditions involved an initial denaturation at 95 °C for 5 min, followed by 40 cycles of denaturation at 95 °C for 15 s, with annealing and extension at 52 °C for 30 s. All reactions were carried out in triplicate in a volume of 20 µL, containing 5 µM of each primer and 1 µL of a 1/10 dilution of cDNA. The specificity of the qPCR amplification products was verified using melt curve analysis. Gene expression was assessed using the 2^−ΔΔCt^ method in Microsoft Excel (2024). This method calculated the relative expression of the target gene after normalizing the three reference genes and comparing it to the non-injected control group using a one-way ANOVA with Tukey’s test in R version 4.1.2.

### 2.6. Evaluation of Tick Fitness

To assess tick fitness, the following parameters were evaluated: number of viable engorged females recovered, engorged female weight, weight of egg mass, and hatching rate. Engorged female tick recovery was assessed by observing the number of viable female ticks recovered after acquisition feeding and comparing the number of those recovered to the number of ticks originally placed (130 ticks per group). Forty engorged females per group (dsRm24, dsControl, and non-injected) were weighed individually and placed into 24-well plates at 26 °C in 96% relative humidity for oviposition. Egg masses laid by the 40 individual females were collected and weighed 21 days post-engorgement, placed into vials and kept at 26 °C for hatching to larvae. Approximately 100 eggs from each individual female egg mass were collected separately into Cell Lysis Buffer (Qiagen) for DNA extraction. Another portion of eggs was spread onto double-sided tape in 24-well plates and sealed with an air permeable cover to assess fecundity through hatching rates by counting the number of successfully hatched larvae vs. total eggs plated.

### 2.7. Infection Rate of B. bovis in R. microplus Adult Females, Egg Mass, and Larvae

Engorged female ticks that were fed on a *B. bovis*-infected calf were used to evaluate hemolymph infection. The engorged females were incubated at 26 °C in 96% relative humidity for oviposition. A portion of the eggs from the individual female ticks was tested for *B. bovis* infection. The other portion of the eggs was incubated at 26 °C in 96% relative humidity to hatch to larvae. The presence of *B. bovis* in adult female ticks was assessed using hemolymph testing on the dsRM24, dsControl, and non-injected groups (n = 40 per group). As previously described [19,22], a hemolymph sample was collected from individual ticks on day 8 post-engorgement by cutting a small portion from the fourth leg and placing the sample onto a glass slide. The samples were then stained using the HEMA 3 Staining System (Thermo Fisher Scientific) and analyzed for the presence of *B. bovis* kinetes under light microscopy. To monitor *B. bovis* presence in the eggs, nested PCR using two sets of ksp-specific primers (Table S1) was performed. Approximately 100 eggs from each of the 40 ovipositing *R. microplus* females were tested (about 4000 eggs), and genomic DNA was extracted using the Puregene DNA extraction kit (Qiagen).

The nested PCR was conducted using JumpStart™ REDTaq^®^ ReadyMix™ Reaction Mix (Sigma Aldrich, Saint Louis, MO, USA) in a 20 µL reaction volume. Reaction 1 of the nested PCR utilized the egg genomic DNA as a template and ksp external primers with an annealing temperature set at 60 °C. Reaction 2 used 0.5 µL of the PCR product generated in reaction 1 as a template, and internal ksp primers also at an annealing temperature of 60 °C. The *R. microplus* α-tubulin gene primers (Table S1) were used to test for intact DNA by PCR. To determine larval infection, a temperature stimulation protocol was carried out on all larvae from 40 females in each group. During this stimulation, the larvae were placed in a 15 °C incubator for 4 days, then moved to a 37 °C incubator for an additional 4 days before being frozen at −20 °C for three days [23]. For genomic DNA extraction and larval testing, approximately 40 individual egg clutches were used. From each clutch, larvae were tested in triplicate, with each replicate consisting of a pool of 30 larvae. This resulted in a total of 90 larvae tested per egg clutch and approximately 120 replicate pooled samples per experimental group to determine vertical transmission. Genomic DNA from each pooled replicate was extracted using the previously described protocol and analyzed via nested PCR using the ksp primer sets. The *R. microplus* α-tubulin gene primers were used to test DNA quality.

**Figure 1:**
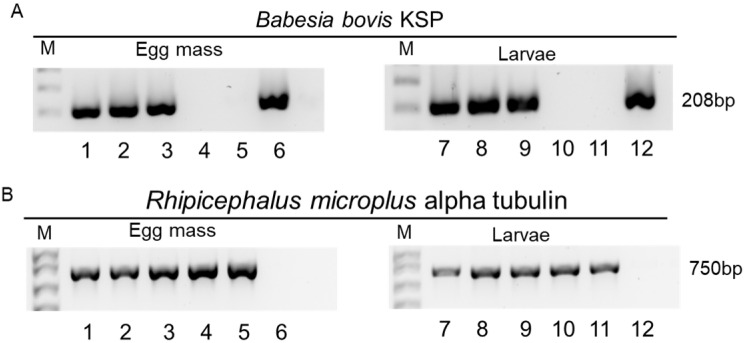
Detection of transovarial transmission of *B. bovis* to egg masses and larvae by PCR assays. (**A**) Detection of *Babesia bovis* infection in egg masses and larvae. (**B**) Amplification of *R. microplus* α-tubulin gene for DNA isolation quality control. On the right side: the expected band size for *B. bovis* ksp and *R. microplus* α-tubulin. The same samples were used to detect genomic DNA from parasites and ticks isolated from egg masses or larvae. Samples 1–5 represent a subset of genomic DNA isolated from egg masses, while sample 6 consists of DNA isolated from a *B. bovis*-infected bovine. Samples 7–11 represent another subset of genomic DNA isolated from larvae, and sample 12 is also DNA extracted from a *B. bovis*-infected bovine. M: 1 kb molecular marker.

To assess transovarial transmission of *B. bovis*, we employed a nested PCR targeting the *Babesia* ksp gene to determine infection status in egg masses and larvae (Figure 1A). For quality control of the extraction method, tick DNA was detected using PCR with primers targeting the *R. microplus* α-tubulin gene. Any samples that did not show the presence of tick DNA were excluded from the analysis (Figure 1B).

### 2.8. Statistical Analyses

All statistical tests were performed in R version 4.1.2 and Prism-GraphPad 6 software was used for data visualization. Gene expression analysis, recovery of engorged ticks, weights of engorged females, and egg mass weights were compared by one-way ANOVA with Tukey’s test. Infection rates of the adults, eggs and larvae were all compared using Chi-square tests with Bonferroni’s correction.

## 3. Results

### 3.1. Proteomic Analysis

Proteomic analysis identified 18 proteins that were significantly differentially expressed (Table 1) between unexposed and *B. bovis*-exposed *R. microplus* female ticks (*p* < 0.05), with four being upregulated in the midgut during *B. bovis* infection, and the rest being down regulated. The differential expression was evaluated by analyzing the fold changes in peptide abundance between female ticks that were unexposed and those exposed to *B. bovis*. Of the four upregulated proteins, only Rm24 was predicted to possess a signal peptide, transmembrane regions (InterPro 103.0 https://www.ebi.ac.uk/interpro/ accessed on 3 April 2025), and fit the predetermined quality criteria of a SEQUEST score greater than 100 with percent coverage exceeding 50% (Table 1). The proteins differentially expressed in response to *Babesia* infection were characterized by molecular function and biological processes (Table S2) using UniProt (https://www.uniprot.org/ accessed on 3 April 2025), Panther Classification System (https://pantherdb.org/ accessed on 3 April 2025), and InterPro (https://www.ebi.ac.uk/interpro accessed on 3 April 2025).

### 3.2. RNA Interference

**Figure 2:**
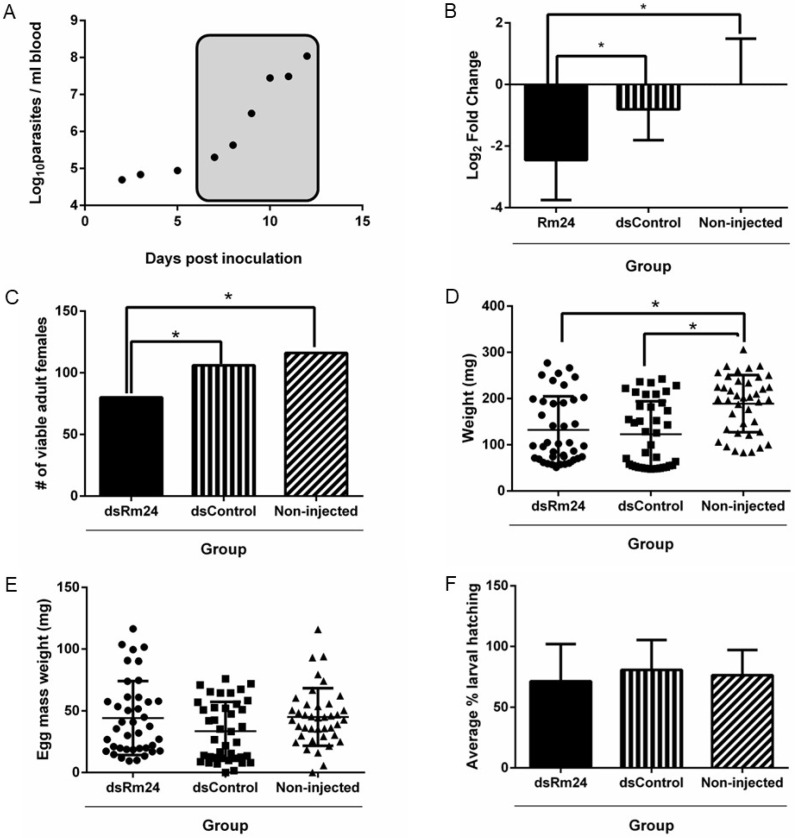
The effect of RNAi on tick fitness. (**A**) Tick feeding during acute infection. The parasitemia was determined by quantitative real-time PCR targeting the ksp gene. Tick feeding window denoted by the box. (**B**) Silencing of the Rm24 gene in tick midguts. The data are represented by the fold-change value (2^−ΔΔCt^) transformed by log base 2. Groups were compared using one-way ANOVA with Tukey’s test. * Indicates *p* < 0.05. Significant differences were observed between the dsRm24 group and the dsControl group and between the dsRm24 group and the non-injected group. (**C**) Number of female *R. microplus* ticks recovered. Comparisons were made by Chi-square tests with Bonferroni corrections. * Indicates *p* < 0.05. Significant differences were observed between the dsRm24 group and the dsControl group and between the dsRm24 group and the non-injected group. (**D**) Engorgement weights of adult female ticks. Groups were compared by one-way ANOVA and Tukey’s test. * Indicates *p* < 0.05. Significant differences were observed between dsRM24 and the non-injected group and between the dsControl group and the non-injected group. (**E**) Egg mass weight. No significant differences were observed between the groups. (**F**) Average percentage of larval hatching per treatment group. Percentage larval hatching was determined by observing the hatched larvae from approximately 100 eggs per clutch from each group. No significant differences were observed between groups.

Based on the proteomic data, we selected the Rm24 upregulated protein to assess functionality in *B. bovis* infection through an RNA interference experiment. The Rm24 gene was silenced significantly with a fold change of 3.7 times when compared to the non-injected control. When compared to the dsControl group, the dsRm24 group exhibited silencing with a fold change of 3.1 times. This represented a knockdown of approximately 62% of Rm24 in the midguts compared to the dsControl group (Figure 2B). There was no significant silencing of Rm24 between the non-injected control and the dsControl groups.

### 3.3. Effect of Gene Silencing on Tick Fitness

In the dsRm24 group, 80 engorged female ticks were recovered at the end of the acquisition feed compared to 106 ticks in the dsControl group and 116 ticks from the non-injected group (Figure 2C). These recovery rates demonstrate that the silencing of Rm24 significantly decreases survivability. The average weight of engorged females in the non-injected control group was 188.9 mg ± 61.1 mg, whereas in the dsControl group, the mean engorgement weight was 122.7 mg ± 70.4 mg, and in the dsRm24 group, it was 132.2 mg ± 71.7 mg, demonstrating a significant decrease in weight for those groups that were injected, *p* < 0.05 (Figure 2D). There was no significant difference between the dsRm24 and dsControl groups. Silencing of Rm24 had no effect on egg mass weight with the dsRm24 group having an average egg mass of 44.2 mg ± 27.6 mg compared to the dsControl group with an average egg mass of 32.8 mg ± 23.8 mg and the non-injected group with an average of 44.2 mg ± 27.6 mg (Figure 2E).

Hatching rates were also unaffected by Rm24 silencing, confirmed by no statistical differences in larval hatching recorded between groups. In the two control groups, non-injected and dsControl, hatching rates were 76% and 80%, respectively, whereas the dsRm24 group had a hatch rate of 71% (Figure 2F).

### 3.4. Babesia Bovis Infection of Engorged R. microplus Female Ticks, Egg Mass, and Larvae

**Figure 3:**
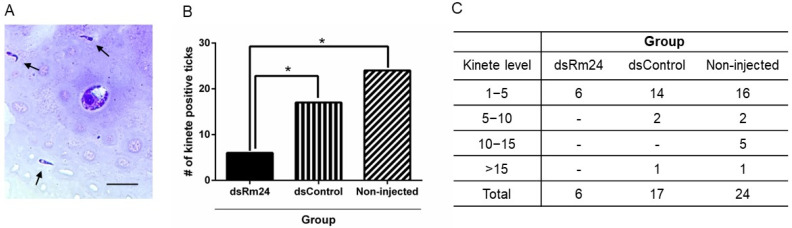
Infection of female ticks with *B. bovis* kinetes. (**A**) Hemolymph smear containing kinetes visualized with light microscopy, scale bar: 25 µm, (**B**) The number of kinete-positive adult female ticks in each treatment group; the dsRm24 group and control groups were compared by Chi-square tests with Bonferroni correction. * Indicates *p* < 0.05. (**C**) Level of *B. bovis* kinetes in the hemolymph. Data are presented as the number of female ticks with having *B. bovis* kinetes in their hemolymph.

To determine *B. bovis* infection status of adult female ticks that fed on an infected calf during ascending *B. bovis* parasitemia, 40 engorged female ticks were used for hemolymph testing. The presence of kinetes in tick hemolymph was determined by light microscopy (Figure 3A). Overall, silencing of Rm24 impacted acquisition of *B. bovis* in female ticks. In the dsRm24 group, only 15% of ticks tested positive for kinetes during the hemolymph testing, whereas the dsControl and non-injected groups showed kinetes in 42.5% and 60%, respectively (Figure 3B). There were significant differences between dsRm24 and the control groups *p* < 0.05. Not only did more ticks have kinetes in their hemolymph, but there were more kinetes present (Figure 3C).

**Figure 4:**
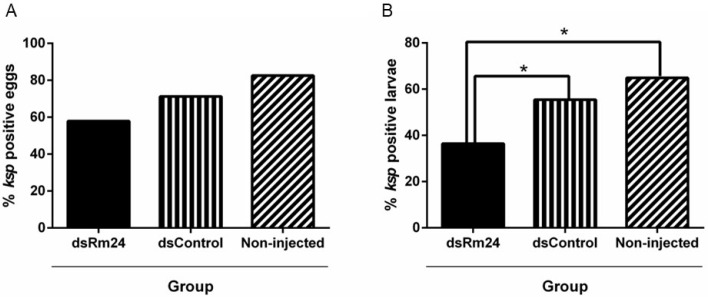
Detection of *B. bovis* in egg masses and larvae. (**A**) Infection rate of *R. microplus* eggs from Rm24 silenced adult female ticks and controls. The percentages of infection determined by nested PCR were compared using Chi-square tests with Bonferroni correction although no significant difference was noted. (**B**) Infection rate of *B. bovis* in larval progeny from Rm24 silenced *R. microplus* female ticks. The percentages of infection determined by nested PCR in larval progeny were compared using Chi square tests with a Bonferroni correction. * Indicates *p* < 0.05.

Fewer egg masses from female ticks in the dsRm24 group tested positive for *B. bovis* (57.9%) than from the dsControl group (71.2%) and the non-injected group (82.5%), although the difference was not significant (Figure 4A). The larvae pools from female ticks in the dsRm24 group showed significantly lower infection rates than the control groups, with only 36.4% of the larvae pools being positive for infection with *B. bovis*, whereas the dsControl group had an infection rate of 55.4% and the non-injected group had an infection rate of 64.9%, *p* < 0.05 (Figure 4B).

## 4. Discussion

In this study, we demonstrated the impact of *B. bovis* on the midgut proteome of *R. microplus*, leading to the identification of 18 differentially expressed proteins during *B. bovis* infection. We further classified these midgut proteins based on their up or downregulation, predicted protein domains, and biological or molecular functions. Our findings support previous research indicating that *Babesia* infections significantly affect protein expression in biological vectors [24,25,26]. Notably, a prior study reported that *B. bovis* gamete development occurs in the tick midgut around days 3 and 4 post-engorgement [27], which may play a crucial role in altering protein expression in midgut epithelial cells. Consistent with Rachinsky et al. (2008), we identified both upregulated and downregulated tick proteins in response to *B. bovis* [24]. However, the specific proteins and their functions differed from those in Rachinsky et al. (2008) [24]. The proteins identified in the previous study have a range of biological functions including metabolic enzymes, aldehyde dehydrogenases, actin, lamin, prohibitin-like proteins, ribophorin I, and ribosomal proteins. The downregulation of heat shock proteins was also observed in this present study and the previous study by Rachinsky et al. (2008) [24]. These discrepancies can be attributed to the varied methodologies used to assess differential expression as well as advancements in technology. The proteins identified in our study align with reports from other studies (Table S2), including the downregulation of metabolism and immune system-related arthropod proteins during infection [28,29,30,31,32,33,34,35,36,37,38,39,40,41,42,43,44,45,46,47,48,49,50].

Several downregulated proteins were identified as involved in metabolic processes. A recent study by Earls et al. (2025) has shown that across *R. microplus* developmental stages, infection with *B. bovis* impacts metabolic rates [28]. In the replete adult female infected with *Babesia*, metabolic rates were significantly lowered. It has been postulated that this downregulation of metabolism may inhibit genes related to the innate immune response, allowing parasites to more efficiently infect and colonize the tick’s organs [28,29]. Dysregulation of metabolism to impact the host immune response has been seen in other pathogens including *Anaplasma phagocytophilum* which down regulates metabolism in *Ixodes scapularis* during infection [30].

Further, we also identified proteins involved in proteolysis, such as metallopeptidases and legumain-like proteases. In ticks, both metallopeptidases and cysteine proteases are crucial for blood feeding and digestion, again playing a role in the metabolic processes in ticks which have been tied to the modulation of the innate immune response [31,32]. We also identified downregulated proteins that function as protease inhibitors and were described as cystatins and Kunitz domain-containing proteins. Cystatins are associated with a broad range of biological processes from modulation of the mammalian immune response to aiding in blood digestion; however, it has also been shown that some cystatins play a crucial role in tick innate immunity [33]. In addition, Zhou et al. (2006) demonstrated that cystatins in *H. longicornis* were significantly induced during blood feeding and that these proteins also directly inhibited the growth of *B. bovis* in vitro [34]. Similarly, it has also been shown that the inhibition of kunitz-domain-containing proteins resulted in increased infection of the tick vector by *Rickettsia* species [35]. Inhibition of the tick’s innate immune system, possibly achieved by the downregulation of key immune proteins, would create a more favorable environment for the infection and colonization of *B. bovis* within the tick midgut.

Likewise, proteins that are classified as heat shock proteins or proteins involved in oxidative stress responses were noted as downregulated. These families of proteins are often described as protective for the tick vector and work to restore homeostasis under stress [36,37,38]. The dysregulation of these responses by pathogen infection has been previously recorded in tick species infected with *Anaplasma* spp. [39]. This relationship may further play into the balance needed to maintain an environment that is accessible to the *B. bovis* parasite but does not detrimentally impact the tick vector. The proteins identified as upregulated in our study were also consistent with previous work that documented the effects of pathogen infection on blood feeding, digestion, gene expression, and gene regulation [31,48,49,50]. Several proteins were described as involved in methylation and metal ion binding. Both methylation and metal ion-binding proteins can play a role in gene regulation and have also been shown to be impacted during pathogen infection [46].

Of the differentially regulated proteins, only one, Rm24, met our predetermined mass spectrometry criteria. Rm24 was predicted to contain a signal peptide and a transmembrane region, suggesting that this protein is surface exposed on midgut epithelial cells and may serve as a potential receptor for *B. bovis* infection. In addition to its preliminary characterization, Rm24-related proteins in other blood-feeding arthropods have been noted as being upregulated during infection with other pathogens. In a study looking at the effects of the protozoan parasite, *Trypansoma cruzi* and the impacts of infection on the proteome of the triatomine insect, it implicated a protein structurally homologous to Rm24 as important to the gut infection of the arthropod vector [51]. In another study, a small, secreted midgut protein with similar structure to Rm24 was also identified as upregulated during infection with *Borrelia burgdorferi* in *Ixodes scapularis* and shown to be crucial in bacteria colonization within the tick [52]. Although Rm24 has not yet been physiologically characterized, we functionally assessed its role in *R. microplus* during *B. bovis* infection by knocking down the Rm24 gene.

Using RNAi, we successfully reduced Rm24 expression, observed a significantly lower number of viable female ticks, and the engorged female ticks weighed less than those in the non-injected control group. Regarding engorgement weight, these results suggest that the injection process may adversely affect tick fitness by wounding the tick cuticle, which aligns with previous findings concerning the effects of microinjection on engorgement weights [53]. Alternatively, since the biological function of Rm24 has yet to be defined, knocking down the Rm24 gene may disturb tick physiology, including digestion and midgut cell integrity, which interfere with the female tick’s ability to acquire a bloodmeal and survivability. This has been previously noted with other midgut proteins, such as Bm86, where its abrogation resulted in disruption of midgut cell integrity, leading to dysregulation of blood feeding and digestion [54,55].

Furthermore, we found that the dsRm24 group showed significantly lower infection rates in engorged female ticks, suggesting that Rm24 is important for *B. bovis* to infect the midgut of *R. microplus*. We employed a HEMA3-stained hemolymph smear technique, which involved removing a single distal leg segment and blotting approximately 0.2 µL of the exuding hemolymph onto a slide. We also noted a lower number of egg masses that tested positive via nested PCR, showing a trend toward reduced *B. bovis* infection in the egg masses from engorged female ticks injected with dsRm24, although the differences were not statistically significant. The higher ratio of infected egg masses detected by nested PCR compared to the ratio of infected engorged females identified through hemolymph testing is due to the lower sensitivity assay employed for the detection of kinetes in tick hemolymph. While a stained hemolymph smear cannot match the sensitivity of nested PCR, it allows for testing of the ticks while keeping them viable for oviposition. We also observed a significantly lower infection of *B. bovis* in the larval progeny from the dsRm24 group of adult females based on nested PCR results, further suggesting that the Rm24 protein is critical for *B. bovis* to infect *R. microplus* female ticks. RNAi using microinjection directly into the hemocoel has the potential to affect other organs, impacting *B. bovis* infection in the next tick generation; however, this was not evaluated during this study. Additional experiments are necessary to determine the expression of Rm24 in other tick organs, especially tick ovaries, a key organ for *B. bovis* vertical transmission.

## 5. Conclusions

In conclusion, further investigation into the role of Rm24 during infection is necessary and experiments are needed to disrupt the interactions between *B. bovis* and Rm24. We could not completely block the vertical transmission of *B. bovis* to the next tick generation due to the limitations of RNAi, which cannot fully eliminate gene expression. It is also possible that there are proteins expressed in midgut cells with redundant functions that allow *B. bovis* to continue infecting the midgut. To completely abrogate Rm24 and its functions, further experiments may be conducted by knocking out the Rm24 gene or by developing antibodies to block *B. bovis* and Rm24 protein interactions. Additional research is necessary to identify other midgut proteins with potential redundant functions that enable *B. bovis* to infect midgut epithelial cells. Our data indicate that Rm24 is important for the infection of *B. bovis* in the midgut. This finding is supported by our RNAi results, which showed significantly lower infection rates in adult female ticks after Rm24 was knocked down. This reduction in Rm24 not only decreased the infection in the adult females but also led to significantly fewer infected larval progeny. Therefore, the Rm24 protein could be a promising target for future strategies to prevent the spread of *B. bovis* via tick transmission.

## Figures and Tables

**Table 1:** Tick midgut proteins differentially regulated upon *Babesia bovis* infection.

Accession	Protein Description	Sequest HT Score	Coverage %	Sum PEP Score	# Peptide	# PSMs	Up/Down Regulation	*p* Value
A0A6M2D867	Conserved secreted	419.11	62	68.00	9	132	2.1-fold increase	0.022
A0A6G5AC63	Selenium binding	424.94	61	149.80	21	138	2.3-fold increase	0.008
A0A6M2CI31	Farnesoic acid o-methyltransferase	83.32	87	49.43	8	29	11.5-fold increase	0.049
A0A6G5A0W4	Metallopeptidase	55.28	25	29.37	6	15	3.3-fold increase	0.048
A0A6G4ZX34	Myosin class ii heavy chain	171.62	27	69.24	14	44	1.5-fold decrease	0.041
A0A034WTW0	Kunitz domain-containing protein	308.11	24	61.48	11	97	1.3-fold decrease	0.034
A0A6G5A749	cystatin	75.3	51	19.71	5	25	3.8-fold decrease	0.027
A0A6M2CKK4	Calmodulin	71.47	26	24.96	2	17	3.3-fold decrease	0.035
A0A6M2CYW0	Proline and glutamine-rich splicing factor	65.99	10	29.64	5	18	3.0-fold decrease	0.031
A0A6M2CKP6	Phosphoenolpyruvate carboxykinase (GTP)	264.53	40	94.60	15	70	1.9-fold decrease	0.006
A0A6M2CGM2	Legumain-like protease	58.15	21	26.82	4	16	4.9-fold decrease	0.022
A0A6G5A5X8	Protein quiver	39.38	12	14.64	2	11	9.0-fold decrease	0.049
A0A6M2CJA6	Plasma membrane glycoprotein	39.21	12	15.41	4	11	3.3-fold decrease	0.032
A0A6M2CM57	Phosphoglucomutase phosphomannomutase	24.19	11	14.49	4	7	3.0-fold decrease	0.023
A0A6G4ZZC0	metalloprotease m41	23.56	8	18.94	3	7	2.9-fold decrease	0.046
A0A6G5ADT3	transcriptional regulator	14.42	13	9.49	2	5	5.8-fold decrease	0.022
Q2XW15	Glutathione peroxidase	13.94	29	14.391	3	6	10.2-fold decrease	0.043
A0A6M2D080	Molecular chaperone	10.26	9	5.591	2	3	9.8-fold decrease	0.047

PEP: posterior error probability; PSM: Peptide Spectrum Match; #: number of; SP: signal peptide; TM: transmembrane region.

## Data Availability

The original contributions presented in this study are included in the article/Supplementary Material. Further inquiries can be directed at the corresponding author.

## Supplementary Materials

The following supporting information can be downloaded at https://www.mdpi.com/article/10.3390/microorganisms13081713/s1, Table S1. Primers targeting *B. bovis* and *R. microplus* utilized in the study. bp: base pairs. Table S2. Classification *R. microplus* midgut proteins regulated in response to *Babesia bovis* infection.
