# Supplementary material for: Identification of a Tick Midgut Protein Involved in Babesia bovis Infection of Female Rhipicephalus microplus Ticks
Source: Microorganisms. 2025 Jul 22;13(8):1713. doi: 10.3390/microorganisms13081713 (PMC12388509; doi:10.3390/microorganisms13081713)
Supplement: Supplementary file 1 [file microorganisms-13-01713-s001.zip › microorganisms-3729164-supplementary.pdf]

Table S1. Primers targeting *B. bovis* and *R. microplus* utilized in the study.

Primer	Forward primer (5′-3′)	Reverse primer (5′-3′)	Product size
BbovKSP	GGGCAATGTTAATGGCAAGATAG	CCTCAACCTCAGCCTCAATAAG	116 bp
RmGADPH	GTGAACCACACCACCTACAA	AGTTGTCGTGGATGACCTTG	100 bp
RmELF1A	CTGCAAGTTCAGGGAGATCAA	TTGGAAGGTACCAGGTCAATG	117 bp
RmS3a	CAGGAACATTGGCAAGACCT	GGCTTGGGAGACTTCAAACA	94 bp
RmRm24	TGAGAAGAGCTACGAGCAAGA	TAGCCAAGGTTGGTGAGGTA	78 bp
Rmtubulin	CTCGATCCTGACCACTCACA	GGGACACCAGTCGACAAACT	750 bp
BbovKSPExt	TGCGATTGCTGCTGCTAAGA	AGCAGCTGTAGCAACCATGT	547 bp
BbovKSPInt	GCCACCGCTGTCAATGTAAA	ACATCCTAATGGCACGGACG	208 bp

Table S2. Classification *R. microplus* midgut proteins regulated in response to *Babesia bovis* infection.

UniProt Accession	Description	Functional classification
**A0A6M2D867**	Conserved secreted protein	This family of proteins is functionally uncharacterized.
**A0A6G5AC63**	Selenium binding protein	Molecular Function: Selenium binding. Biological Process: regulates free selenium levels in the cell, plays a key role in gene expression regulation and pathogen response [48,49].
**A0A6M2CI31**	Farnesoic acid O-methyltransferase	Molecular Function: Methyltransferase activity. Biological Process: biosynthesis of juvenile hormones in insects which is crucial for their development [50].
**A0A6G5A0W4**	Metallopeptidase	Molecular Function: uncharacterized. Biological Process: essential for blood feeding and digestion in blood feeding arthropods [31].
**A0A6G4ZX34**	Myosin class II heavy chain	Molecular Function: actin filament binding, ATP binding, microfilament motor activity. Biological Process: Class-II myosins are finely regulated by the phosphorylation of the myosin light chain and the binding of calcium ions (Ca2+), facilitates interactions needed for movement. May be essential for feeding and the dynamics of the midgut during the post-repletion phase [40].
**A0A034WTW0**	Kunitz domain-containing protein 1	Molecular Function: Serine-type endopeptidase inhibitor activity. Biological Process: Suggested that it is involved in strong innate immune response in plants, invertebrates, and possibly arthropods [35].
**A0A6G5A749**	Cystatin	Molecular Function: Cysteine-type endopeptidase inhibitor activity, protease inhibitor, thiol protease inhibitor, endopeptidase inhibitor activity. Biological Process: Crucial for tick immunity and effectively inhibits the growth of *B. bovis* in vitro, underscoring its significance in disease management [34].
**A0A6M2CKK4**	Calmodulin	Molecular Function: Calcium ion binding, carbohydrate kinase activity, phosphotransferase activity. Biological Process: Hexose metabolic processes, cell signaling, and their significance in blood feeding among ticks [41].
**A0A6M2CYW0**	Proline and glutamine-rich splicing factor	Molecular Function: RNA-binding. Biological Process: Regulation of DNA-templated transcription
**A0A6M2CKP6**	Phosphoenolpyruvate carboxykinase (GTP)	Molecular Function: GTP binding, Phosphoenolpyruvate carboxykinase (GTP) activity, transition metal ion binding carboxy-lyase activity. Biological Process: Metabolic processing, cellular response to glucose stimulus, gluconeogenesis, response to lipids, fatty acid catabolism, response to starvation, cellular response to oxygen-containing compounds. Involved in the immune response to pathogen infections in insects and arthropods [29,30].

**A0A6M2CGM2**	Legumain-like protease	Molecular Function: Cysteine-type endopeptidase activity, hydrolase, protease. Biological Process: Catabolism of proteins, involved in vacuolar protein processing which plays a role in blood feeding and digestion in ticks [32,41].
**A0A6G5A5X8**	Protein quiver	Molecular Function: Unknown. Biological Process: The regulation of synaptic transmission, particularly through cholinergic signaling, is crucial for sleep in Drosophila. Essential for maintaining sleep homeostasis under both normal conditions and after sleep deprivation [42,43].
**A0A6M2CJA6**	Plasma membrane glycoprotein	Molecular Function: Scavenger receptor activity. Biological Process: Pattern recognition receptors play a crucial role in innate immunity [44].
**A0A6M2CM57**	Phosphoglucomutase/ phosphomannomutase	Molecular Function: Magnesium ion binding, phosphopentomutase activity, isomerase, intramolecular transferase activity. Biological Process: Drives essential biochemical reactions during metabolic processing which supports the blood-feeding mechanisms of ticks [45].
**A0A6G4ZZC0**	Metalloprotease	Molecular Function: ATP binding, ATP hydrolysis activity, ATP-dependent peptidase activity, metalloendopeptidase activity, zinc ion binding, hydrolase, metalloprotease. Biological Process: Mitochondrial protein processing and proteolysis play important roles in blood feeding and digestion in ticks [31].
**A0A6G5ADT3**	Transcriptional regulator	Molecular Function: Unknown. Biological Process: Glycolate biosynthesis, glyoxal metabolism, response to oxidative stress, and the protective response of arthropod vectors to restore homeostasis under stress [37].
**Q2XW15**	Glutathione peroxidase	Molecular Function: Peroxidase activity, oxidoreductase, peroxidase, antioxidant activity. Biological Process: Associated with the response to oxidative stress, representing the protective response of arthropod vectors to restore homeostasis under stress [37,38].
**A0A6M2D080**	Molecular chaperone	Molecular Function: ATP binding, Hsp70 protein binding, metal ion binding, unfolded protein binding. Biological Process: Associated with protein folding, heat response, protein stabilization, and the protective response of arthropod vectors to restore homeostasis under stress [36].
